# Comprehensive Analysis of the Functions and Prognostic Value of RNA-Binding Proteins in Thyroid Cancer

**DOI:** 10.3389/fonc.2021.625007

**Published:** 2021-03-17

**Authors:** Yue Ma, Shi Yin, Xiao-feng Liu, Jing Hu, Ning Cai, Xiao-bei Zhang, Li Fu, Xu-chen Cao, Yue Yu

**Affiliations:** ^1^ The First Department of Breast Cancer, Tianjin Medical University Cancer Institute and Hospital, National Clinical Research Center for Cancer, Tianjin, China; ^2^ Key Laboratory of Cancer Prevention and Therapy, Tianjin, China; ^3^ Tianjin’s Clinical Research Center for Cancer, Tianjin, China; ^4^ Key Laboratory of Breast Cancer Prevention and Therapy, Tianjin Medical University, Ministry of Education, Tianjin, China; ^5^ Hepatic Surgery Center, Tongji Hospital, Tongji Medical College, Huazhong University of Science and Technology, Wuhan, China; ^6^ Department of Anesthesiology, Tianjin Medical University Cancer Institute and Hospital, National Clinical Research Center for Cancer, Tianjin, China; ^7^ Department of Breast Cancer Pathology and Research Laboratory, Tianjin Medical University Cancer Institute and Hospital, National Clinical Research Center for Cancer, Tianjin, China

**Keywords:** thyroid carcinoma, RNA-binding protein (RBP), TCGA, gene signature, prognosis

## Abstract

RNA binding proteins (RBPs) have been proved to play pivotal roles in a variety types of tumors. However, there is no convincible evidence disclosing the functions of RBPs in thyroid cancer (THCA) thoroughly and systematically. Integrated analysis of the functional and prognostic effect of RBPs help better understanding tumorigenesis and development in thyroid and may provide a novel therapeutic method for THCA. In this study, we obtained a list of human RBPs from Gerstberger database, which covered 1,542 genes encoding RBPs. Gene expression data of THCA was downloaded from The Cancer Genome Atlas (TCGA, n = 567), from which we extracted 1,491 RBPs’ gene expression data. We analyzed differentially expressed RBPs using R package “limma”. Based on differentially expressed RBPs, we constructed protein-protein interaction network and the GO and KEGG pathway enrichment analyses were carried out. We found six RBPs (AZGP1, IGF2BP2, MEX3A, NUDT16, NUP153, USB1) independently associated with prognosis of patients with thyroid cancer according to univariate and multivariate Cox proportional hazards regression models. The survival analysis and risk score analysis achieved good performances from this six-gene prognostic model. Nomogram was constructed to guide clinical decision in practice. Finally, biological experiments disclosed that NUP153 and USB1 can significantly impact cancer cell proliferation and migration. In conclusion, our research provided a new insight of thyroid tumorigenesis and development based on analyses of RBPs. More importantly, the six-gene model may play an important role in clinical practice in the future.

## Introduction

Cancers are now the leading cause of death worldwide, and thyroid cancer is one of the top 10 cancer types with about 567,233 new cases and 41,071 deaths annually (1). The common subtypes of thyroid cancer based on histological characteristics are anaplastic, follicular, medullary, and papillary thyroid carcinoma (2). The age-standardized 5-year relative survival rate of thyroid cancer can reach 85%, which is recognized as an excellent vital prognosis (3, 4). However, the incidence of thyroid cancer continued to increase in many countries over the last decades (5), along with increased use of diagnostic imaging and surveillance (2). Despite the well-developed surveillance system, the newly diagnosed cases still account for a high proportion of cancer incidence. Moreover, patients with advanced-stage thyroid carcinoma have a poor 5-year survival rate. Therefore, accurate detection of thyroid cancer as early as possible is of great concern. This had led to an urgent need for new diagnostic markers and therapeutic targets for THCA, and a systematic study to explore the differentially expressed genes in thyroid cancer is needed to provide new biomarkers.

RNA-binding proteins (RBPs) interact with various kinds of RNA, playing critical roles in post-transcriptional regulation. A catalog of 1,542 experimentally validated human RBPs had been identified by high-throughput screening in diverse human cell types (6, 7). They create functional units by binding to sequence-specific motifs or RNA secondary structures, utilizing their specific RNA-binding domains (8). RBPs can control multiple targets by influencing different post-transcriptional steps including RNA splicing, polyadenylation, stability, localization, translation, and degradation by establishing dynamic interactions with other coding and non-coding RNAs in so-called ribonucleoprotein complexes (9, 10).

Given that RBPs have pivotal functions in post-transcriptional regulation, minor changes in the expressions and/or activities of RBPs may lead to extensive disruptions of downstream regulatory networks. Thus, it is inevitable that alterations in RBPs underlie several pathological conditions. Studies have proved that RBPs show abnormal expression in tumors relative to adjacent normal tissues (11–13). Various RBP regulation mechanisms have been clarified in cancer cells, including genomic alterations, transcriptional and post-transcriptional control, as well as posttranslational modifications (PTMs). In breast cancer, hnRNPM-mediated alternative splicing of CD44 promotes the epithelial-to-mesenchymal transition (EMT) by activating the transforming growth factor-β signaling pathway (TGF-β), which is essential for metastasis (14). Another study showed that huR can bind the mRNA of the apoptosome inhibitor ProTα, improving its stability to increase the protein’s cytoplasmic abundance and translation, eliciting an antiapoptotic program in cervical cancer (15). In glioblastoma, overexpression of RBFOX1 was found to increase the blood-tumor barrier permeability by increasing the stability of MAFF, which promoted doxorubicin delivery across the blood-tumor barrier, resulting in apoptosis of glioma cells (16). In addition to the proto-oncogenic RBPs mentioned above, other studies have also demonstrated the tumor-suppressor roles of many RBPs. HnRNP K haploinsufficiency leads to genomic instability, increased tumor growth, reduced survival, and the development of transplantable hematopoietic neoplasms with myeloproliferation, which indicates that hnRNP K as a tumor suppressor in hematological disorders (17). Sustained KHSRP expression limits the TGF-β-dependent induction of EMT factors and cell migration, while its knockdown can induce the EMT in mammary gland cells (18). However, currently few studies have systematically investigated the predictive roles of RBPs regarding the diagnosis, prognosis, and therapeutic outcomes of thyroid cancer.

In this study, we downloaded the gene expression data and clinical information of patients with thyroid cancer in The Cancer Genome Atlas (TCGA) database. We then screened differentially expressed RBPs and analyzed potential molecular mechanisms using GO term and KEGG pathway enrichment analysis. Univariate Cox regression analysis was used to identify potential prognostic RBPs, which were then used to construct a multivariate Cox proportional hazards regression model for patient prognosis. The nomogram was plotted to predict the patient outcomes quantitatively in practical work. Finally, experimental validation confirmed that downregulation of the RBPs included in the model could significantly affect the proliferation and migration of thyroid cancer cells. This study confirms the significance of RBPs in thyroid cancer, and the RBPs included in the model may be potential biomarkers for diagnosis and prognosis.

## Materials and Methods

### Data Preprocessing and Screening of Differentially Expressed RBPs

Datasets of thyroid cancer were downloaded from The Cancer Genome Atlas (TCGA) (https://portal.gdc.cancer.gov/) database. We obtained gene expression data (FPKM, Fragments Per Kilobase Million) of 58 adjacent normal tissues and 509 tumor tissues, as well as the clinical information of 507 patients. The expression data for a total of 1,491 RBPs were extracted from the raw data using Perl software, and we identified differentially expressed RBPs using the “limma” package with a criterion of |log2 fold change (FC)| ≥ 0.5 and false discovery rate < 0.05 (19). Based on the differentially expressed RBPs, we drew a heatmap and volcano plot using the pheatmap package.

### Construction and Visualization of the Protein-Protein Interaction Network

We used the online database “STRING” (https://string-db.org/) to construct a protein-protein interaction network based on the differentially expressed RBPs. The network was visualized using Cytoscape 3.7.2 software (20), and key subnetworks were screened based on score > 6 and node > 6 using the MCODE (Molecular Complex Detection) app in Cytoscape.

### GO (Gene Ontology) and KEGG (Kyoto Encyclopedia of Genes and Genomes) Functional Enrichment Analyses

To systematically investigate the biological functions of the identified differentially expressed RBPs, we used gene ontology (GO) analysis, including biological process (BP), cellular component (CC), and molecular function (MF). The KEGG database was used to identify potential biological pathways of the differentially expressed RBPs. GO and KEGG pathway analyses were carried out using the R packages clusterProfiler, enrichplot, ggplot2, and org.Hs.eg.db, with a screening criterion encompassing a *p*-value of less than 0.05 and q value (adjusted *p*-value) of less than 0.05.

### Selection of Prognostic RBPs

We evaluated the association between survival duration and the expression levels of the differentially expressed RBPs *via* individual univariate Cox regression analysis using the “survival” package in R. The RBPs with *p*-values of less than 0.05 were considered potential prognostic RBPs.

### Construction and Evaluation of a Prognostic Model

We developed a multivariate Cox proportional hazards regression model to construct a prognostic model for thyroid cancer patients based on the screened prognostic RBPs. The risk score of each patient was calculated as follows:

$$Risk\;Score=\sum_{i=1}^{n}\beta \;i*Expgene(i)$$

where *n* is the number of genes in this model, *Expgene(i)* is the expression level of each gene, and *β* represents the regression coefficient.

To evaluate the performance of this model, we divided patients into high- and low-risk subgroups, based on the median risk score, and the significance of the difference in overall survival (OS) between the two subgroups was calculated *via* Kaplan–Meier (KM) survival analysis using the “survival” package in R and tested using the log-rank test. At the same time, ROC curve analysis was carried out using the “survivalROC” package in R. The *p*-value of survival analysis less than 0.05 and AUC of ROC curve more than 0.7 were considered to be a moderate model. After verification, the model was found to be reliable and could effectively predict the outcomes of THCA patients. Additionally, we drew a nomogram plot to forecast the OS using the rms package in R.

### Functional Enrichment Analysis of Individual Genes

Gene-set enrichment analysis (GSEA) was used to explore the potential biological pathways in the high- and low-expression groups defined by the thyroid cancer patients’ prognostic signature. KEGG gene sets (v7. 0) and phenotype label (High-Expression vs. Low-Expression) files were generated and loaded into the GSEA software (v4.0.3; Broad Institute, Cambridge, MA) (21). The permutation test was conducted 1,000 times, after which we used the R packages plyr, ggplot2, grid, and gridExtra to integrate the results into a single plot.

### Cell Lines and Culture Conditions

The thyroid cancer cell lines TPC1 and BCPAP used in this study were purchased from the American Type Culture Collection. All cell lines were maintained in Dulbecco’s modified Eagle medium (1640) (HyClone, UT, USA) supplemented with 10% fetal bovine serum (FBS) (Gibco, Gaithersburg, MD) at 37°C in a humidified atmosphere comprising 5% CO_2_.

### Antibodies and Small Interfering RNA (siRNA)

The antibodies used in this study are listed in supplementary Materials and Methods. Sequences of small interfering RNAs (siRNAs) are listed in **Supplementary Table S4**.

### Transient Transfection of Thyroid Cancer Cells

Transient transfection of siRNAs (100 nM) were carried out in 6-well plates using FuGENE HD Transfection Reagent (Promega, Madison, WI, USA). Briefly, cells were seeded at a density of approximately 40,000 cells per cm^2^. Medium was refreshed after 12 h and transient transfection was carried out for 12 h in Opti-MEM Reduced Serum Medium (Thermo Fisher Scientific), followed by 24 h of recovery in media containing 10% FBS. siRNAs targeting NUP153 and USB1 and non-targeting negative control (NC) were purchased from RiboBio (Guangzhou, China).

### Proliferation and Migration Assays

Cell proliferation was assessed using the MTT, plate colony formation and EdU assays, according to the manufacturer’s instruction. Transwell and wound healing/scratch assays were used to evaluate cell migration. Experiments were carried out as described in the **Supplementary Materials and Methods**.

### Western Blot Analysis

Cells were lysed with RIPA lysis buffer (Solarbio, China) with a protease inhibitor cocktail (Roche Molecular Biochemicals, Indianapolis, IN, USA). Proteins were resolved by SDS-PAGE, transferred onto polyvinylidene fluoride membranes (Millipore, Bedford, MA, USA), and incubated with primary antibodies overnight at 4°C, followed by incubation with a horseradish peroxidase-conjugated secondary antibody. The blots were visualized using the enzyme chemiluminescence (ECL) reagent (Millipore, GE Healthcare, USA).

### RNA Extraction and Quantitative Reverse-Transcription Polymerase Chain Reaction (qRT-PCR)

The total RNA of cultured cells was isolated using TRIzol^®^ reagent (Beyotime, Shanghai, China) according to the manufacturer’s instructions. The cDNA templates were prepared using a PrimeScript™ RT reagent kit (Takara, Japan) and 1000 ng of total RNA as template in reaction mixtures comprising 10 μl. Quantitative RT-PCR was performed on a CFX96 Real-Time PCR Detection System (BioRad, USA) using SYBR^®^ Premix Ex TaqTM II (Tli RNaseH Plus) (Takara, Japan). The primer sequences are listed in **Supplementary Table S3**. Real-time PCR reactions were performed in triplicate and all samples were analyzed using the ΔΔCT method. Values were normalized to the internal control GAPDH.

### Statistical Analysis

Data were presented as means ± SD. Student’s t-test (two-tailed) was used to determine the significance of differences between the experimental and control groups. The threshold for significance was set to p < 0.05. Kaplan-Meier survival curves and the log-rank test were used to evaluate the outcomes of patients with THCA with different NUP153 and USB1 expression. All calculations were performed SPSS software (IBM Corp., USA).

## Results

### Differentially Expressed RBPs in Thyroid Cancer

**Figure 1:**
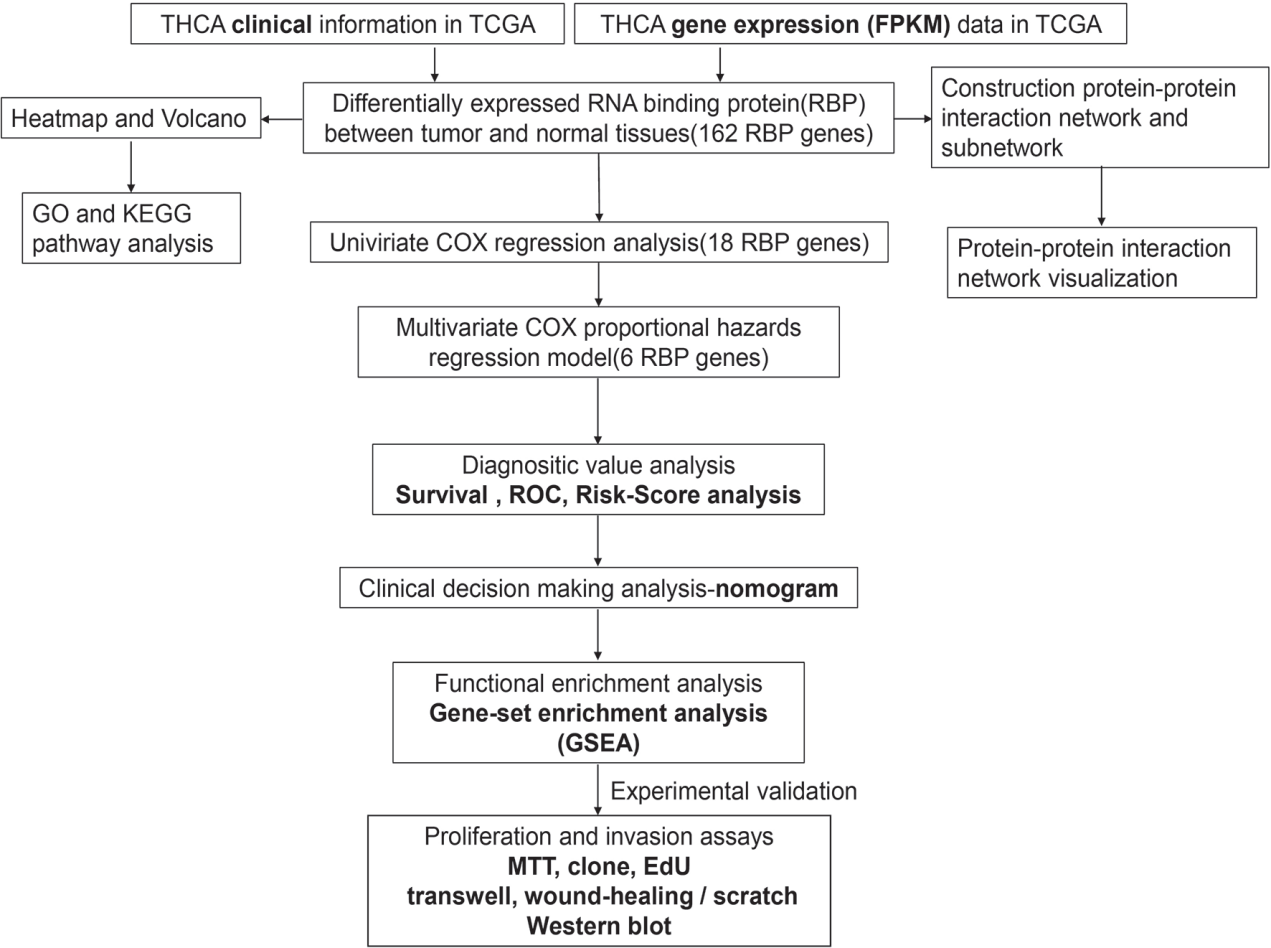
Framework for analyzing the integrated prognostic value of RBPs in thyroid cancer patients based on the TCGA database.

The framework of the analysis applied in this study is shown in **Figure 1**. We downloaded gene expression data (FPKM, Fragments Per Kilobase Million) of 58 adjacent normal and 509 tumor tissues, as well as the clinical information of 507 patients. A total of 1,491 RBPs were extracted from the gene expression data, 162 of which met our inclusion criteria (|log2 fold change (FC)| ≥ 0.5 and false discovery rate < 0.05), including 70 down- and 92 upregulated RBPs. The corresponding heatmap and volcano plot are shown in **Supplementary Figure S1**.

### Protein-Protein Interaction Network

**Figure 2:**
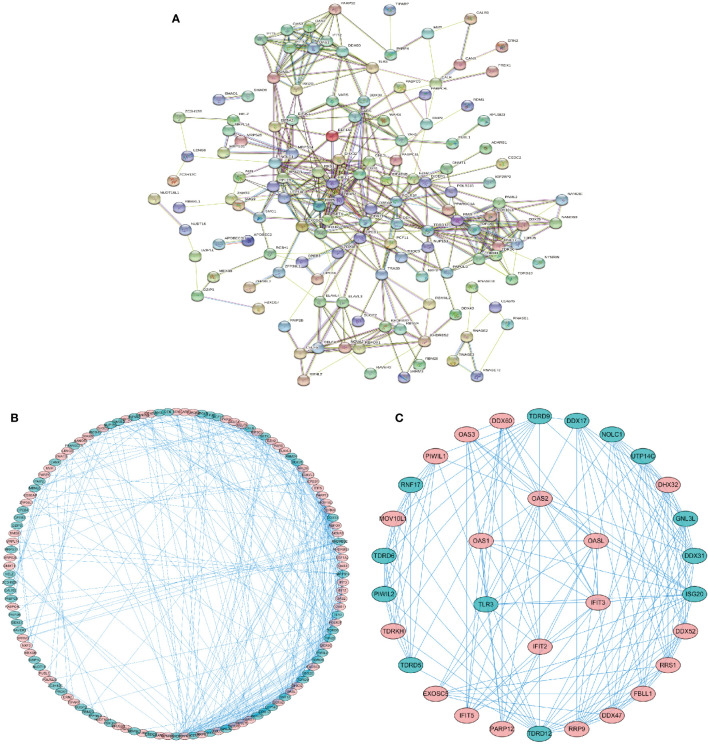
Protein-protein interaction (PPI) network of the identified differentially expressed RBPs. **(A)** PPI network of 162 differentially expressed RBPs. **(B)** Visualization of the PPI network. **(C)** Key subnetworks 1–2 of the PPI network. Subnetwork 1: 26 RBPs, subnetwork 2: 6 RBPs. Red, upregulated RBPs. Green, downregulated RBPs.

We used the online database “STRING” (https://string-db.org) to construct a protein-protein interaction network of the differentially expressed RBPs. The network was visualized by Cytoscape 3.7.2 software to detect potential mechanisms of these differentially expressed RBPs. The PPI network was shown in **Figure 2A**, and the visualized network was shown in **Figure 2B**. It included 138 nodes and 411 edges. We further selected significant sub-networks using the MCODE plug-in and selected the top 2 sub-networks (**Figure 2C**). Subnetwork 1 included 26 nodes and 118 edges, and subnetwork 2 consisted of six nodes and 15 edges. Functional enrichment analysis revealed that: 1. Genes from subnetwork 1 were mainly enriched in ribosome biogenesis, RNA processing, and DNA modification. 2. Genes from subnetwork 2 were significantly enriched in defense response to viral infection, and the type I interferon signaling pathway. GO and KEGG pathway analyses of the two subnetworks are shown in **Supplementary Table S1**.

### Investigation of Biological Pathways Affected by the Differentially Expressed RBPs

**Figure 3:**
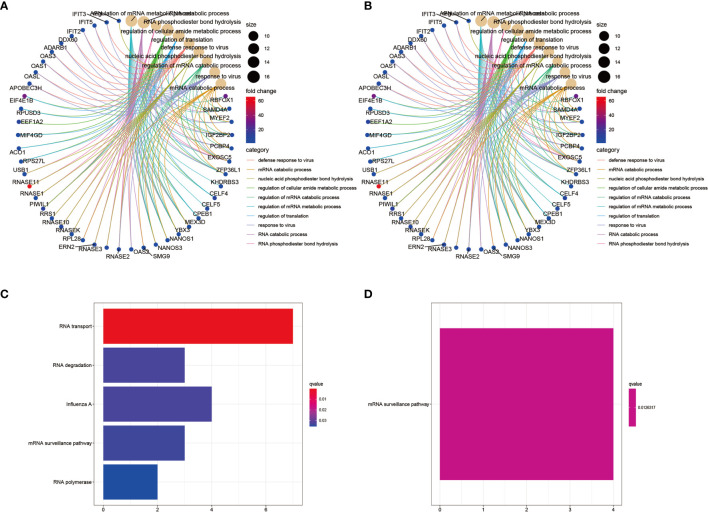
GO and KEGG pathway enrichment analyses of the identified differentially expressed RBPs. **(A)** GO analysis of upregulated RBPs. The result showing the top 10 categories. **(B)** GO analysis of downregulated RBPs. The result showing the top 10 categories. **(C)** KEGG pathway enrichment analysis of upregulated RBPs. **(D)** KEGG pathway enrichment analysis of downregulated RBPs.

To investigate potential biological signaling pathways and molecular mechanisms of the selected RBPs, we analyzed the GO and KEGG enrichment of the up- and downregulated RBPs using the R packages clusterProfiler, enrichplot, ggplot, and org.Hs.eg.db. The top ten GO categories of the up- and downregulated RBPs are shown in **Figures 3A, B**, respectively. The biological processes (BP) analysis showed that the upregulated differentially expressed RBPs were mainly enriched in regulation of RNA metabolic processes, RNA translation, and response to viral infection (**Supplementary Figure S2A** and **Table S2A**). By contrast, the downregulated RBPs were enriched in regulation of translation, regulation of mRNA metabolic processes and RNA splicing (**Supplementary Figure S2B** and **Table S2B**). In terms of cellular components (CC), the upregulated differentially expressed RBPs were notably enriched in cytoplasmic ribonucleoprotein granules and the nucleolar compartment (**Supplementary Figure S2A**). The downregulated differentially expressed RBPs were enriched in cytoplasmic ribonucleoprotein granules, cytoplasmic stress granules and ribosomes (**Supplementary Figure S2B**). Concerning the molecular function (MF), the upregulated differentially expressed RBPs were mainly enriched in catalytic activity-acting on RNA, ribonuclease activity, translation regulator activity, and mRNA 3’−UTR binding (**Supplementary Figure S2A**), while the downregulated RBPs were enriched in helicase activity, catalytic activity-acting on RNA and mRNA 3’−UTR binding (**Supplementary Figure S2B**). The KEGG pathway analysis showed that the upregulated differentially expressed RBPs were enriched in RNA transport, RNA degradation, mRNA surveillance pathways and RNA polymerase function, all related to post-translational regulation related to RNA (**Figure 3C**). The downregulated differentially expressed RBPs were mainly enriched in the mRNA surveillance pathway (**Figure 3D**).

### Screening of Prognostic RBPs

We detected 18 RBPs associating with prognosis by univariate Cox regression and the likelihood-ratio test. Among these, 12 were proto-oncogenic RBPs and six had been recognized as tumor-suppressor RBPs (**Table 1**). A forest plot of the prognostic RBPs is shown in **Supplementary Figure S3**.

**Table 1:** The prognostic effect of prognosis-related RBPs.

Gene	HR^a^	95% CI^b^	*p*-value
PARP12	0.684	0.486–0.962	0.029
NUP153	1.466	1.116–1.927	0.006
TDRD5	2.401	1.133–5.018	0.038
AZGP1	2.236	1.508–3.318	<0.001
TDRKH	0.736	0.545–0.995	0.046
CANX	1.006	1.002–1.010	0.007
RPS27L	0.799	0.674–0.947	0.01
SMAD1	1.992	1.102–3.603	0.023
IGF2BP2	0.915	0.840–0.996	0.039
TDRD6	1.434	1.161–1.771	<0.001
NUDT16	1.101	1.022–1.186	0.011
KHDRBS2	1.260	1.084–1.464	0.003
PPARGC1A	1.139	1.033–1.256	0.009
EEF1A2	1.019	1.005–1.034	0.009
MRPL14	0.961	0.926–0.998	0.039
MEX3A	1.163	1.008–1.342	0.039
USB1	0.734	0.555–0.970	0.03
NOLC1	1.130	1.032–1.236	0.008

^a^HR, Hazard ratio; ^b^CI, Confidence interval.

### Construction and Evaluation of a Prognostic Model

With the screening criteria where survival analysis less than 0.05 and AUC of ROC curve more than 0.7, six genes were incorporated into the multivariate Cox proportional hazards regression model. These were AZGP1, IGF2BP2, MEX3A, NUDT16, NUP153, and USB1. The risk score of each thyroid cancer patient was calculated according to the formula:

$$\begin{array}{l} Risk\;Score=\;(1.021*\;Exp\;AZGP1)\;+\text{ (}-0.09\;*Exp\;IGF2BP2)+\text{ (}0.241\;*Exp\;MEX3A)\\ +\;(0.127\;*Exp\;NUDT16)\;+\;(0.388\;*Exp\;NUP153)\;+\;(-0.236\;*Exp\;USB1) \end{array}$$

**Figure 4:**
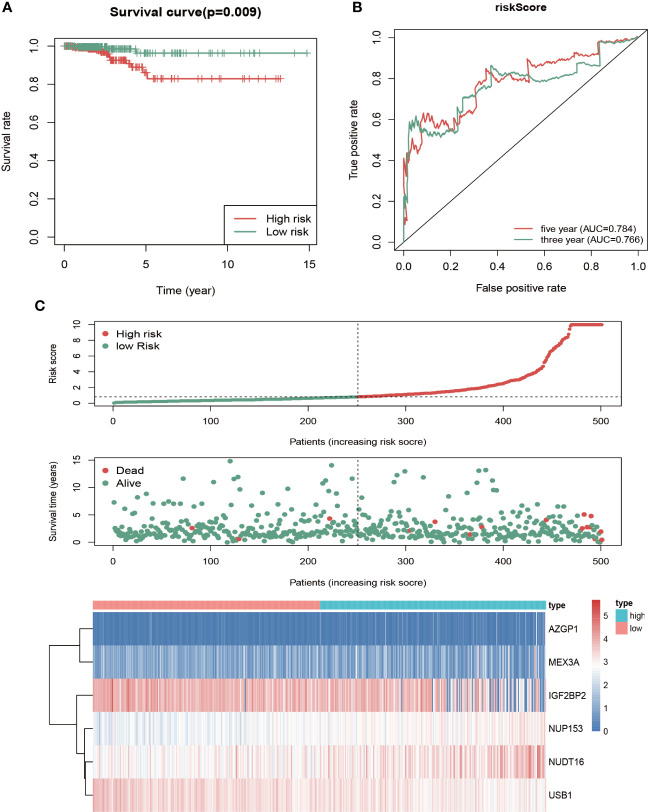
Risk score analysis of the six-gene prognostic model in high- and low-risk groups of THCA patients in TCGA. **(A)** Kaplan-Meier survival analysis of differences between high- and low-risk groups in thyroid cancer showed that the OS was longer in the low-risk group than the high-risk group. Red, high risk. Green, low risk. **(B)** Time‐dependent receiver operating characteristic (ROC) analysis was utilized to evaluate the predictive performance of the model. Green, 3-year (AUC = 0.766). Red, 5-year (AUC = 0.784). **(C)** Risk score distribution (top), survival status distribution (middle) and heatmap of six RBPs (bottom) between high- and low-risk groups of thyroid cancer patients.

To assess this model’s predictive power, patients were divided into high- and low-risk groups based on the median risk score, followed by survival analysis and ROC analysis. The high-risk group showed a significantly lower OS rate compared with the high-risk group (**Figure 4A**). We next conducted ROC analysis to further evaluate the prognostic power of the six-RBP gene model, which revealed AUC values of the ROC curve for OS of 0.766 (3-year OS) and 0.784 (5-year OS) (**Figure 4B**). The risk score duration (**Figure 4C**, top), survival status duration (**Figure 4C**, middle) and expression heatmap of the six biomarkers (**Figure 4C**, bottom) in the high- and low-risk groups were analyzed, which revealed that high-risk patients have a lower survival duration and the gene expression levels are significantly different between the high- and low-risk groups. These results indicated that this model had moderate sensitivity and specificity.

**Figure 5:**
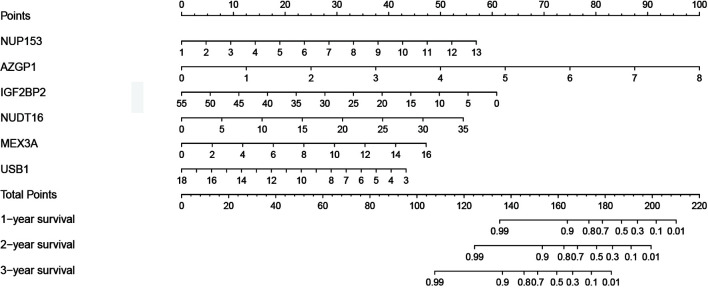
Nomogram for predicting the 1-, 2-, and 3-year OS of thyroid cancer patients in the TCGA cohort using the identified six-gene signature.

To establish a quantitative prognosis for thyroid cancer patients, we integrated the six RBP markers to build a nomogram, which was then used to calculate the estimated survival probabilities of thyroid cancer patients at 1, 2, and 3 years by plotting a vertical line across the total point axis and each prognostic axis (**Figure 5**).

After analyzing the relationship between the expression of the RBPs and prognosis, we next evaluated the prognostic value of different clinical features, including age, gender, T, N, histological type, radiation exposure history, and risk score, in 391 thyroid cancer patients by conducting univariate and multivariate regression analyses. The univariate regression analysis showed that T, radiation exposure history and risk scores were associated with the OS of thyroid cancer patients. However, we found that only T and risk score were independent prognostic factors related to OS according to multivariate regression analysis (**Table 2**).

**Table 2:** The prognostic effect of clinical parameters.

	Univariate analysis			Multivariate analysis		
	HR^a^	95% CI^b^	*p*-value	HR	95% CI	*p*-value
Age	11.40	1.08–78.30	0.998	10.25	2.20–69.78	0.997
Gender	2.67	0.81–8.83	0.107	1.47	0.41–5.20	0.552
Radiation Exposure	5.63	1.20–26.42	0.028	4.28	0.89–20.62	0.070
T	3.99	1.73–9.20	0.001	2.52	1.16–5.50	0.020
N	1.67	0.49–5.72	0.414	0.91	0.26–3.20	0.885
Histological Type	0.01	0.01–1.20	0.998	0.05	0.03–1.03	0.999
Risk Score	1.35	1.15–1.58	<0.001	1.38	1.16–1.64	<0.001

^a^HR, Hazard ratio; ^b^CI, Confidence interval.

### NUP153 Is Involved in the Maintenance of Proliferation in Thyroid Cancer

**Figure 6:**
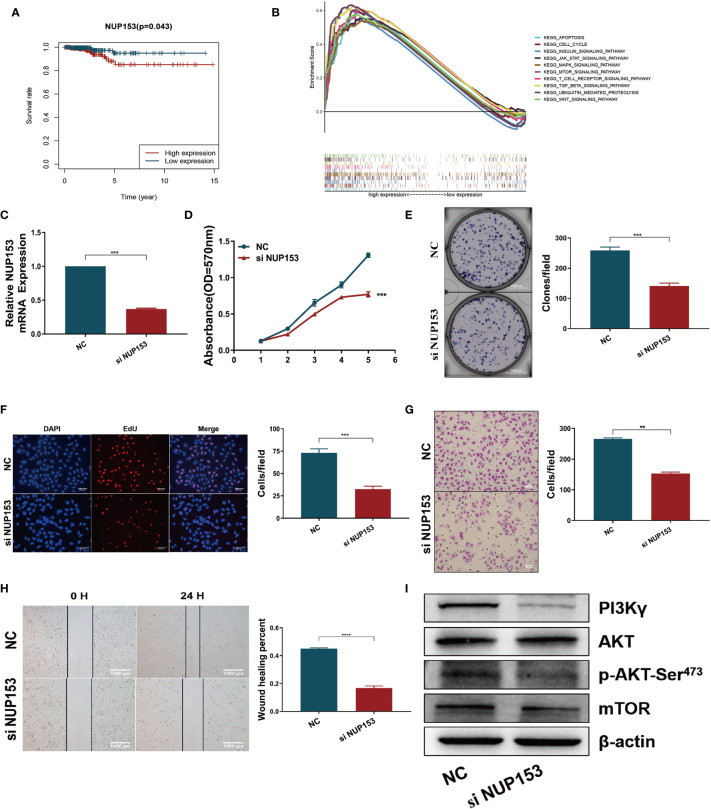
Downregulation of NUP153 inhibited the proliferation and migration of TPC1 thyroid cancer cells. **(A)** Kaplan-Meier survival analysis revealed that high expression of NUP153 was negatively associated with OS in the TCGA cohort. Red, high expression. Blue, low expression. **(B)** Top ten significantly enriched categories in the NUP153-high group compared with its counterpart in THCA. **(C)** Real-time quantitative PCR for the expression of NUP153 in different groups. **(D**–**F)** Growth inhibition in TPC1 cells was determined by MTT **(D)**, colony formation **(E)**, and EdU **(F)** assays. **(G, H)** Cell migration inhibition in TPC1 cells was determined by transwell **(G)** and wound healing/scratch **(H)** assays. **(I)** Western blot validation confirmed decreased expression of PI3Kγ, P-AKT-Ser^473^, and mTOR. ***P < 0.001, **P < 0.01.

After discovering that the expression of NUP153 was higher in the high-risk patients than the low-risk group (**Figure 4C**, bottom), and finding that high expression of NUP153 was negatively associated with prognosis in the survival analysis (**Figure 6A**), we explored the biological signaling pathways differentially enriched between the NUP153 high/low expression groups by gene-set enrichment analysis (GSEA). The results indicated that the PI3K/AKT/mTOR signaling pathway had a significant enrichment score (NES = 2.03, NOM *p* < 0.0001) (**Figure 6B**). Then, we carried out experiment validation. First, we transfected NUP153 specific siRNA into thyroid cancer cell line TPC1 (**Figure 6C**) and BCPAP (**Supplementary Figure S4A**) to decrease the expression of NUP153. The functional experiments showed that the downregulation of NUP153 suppressed the proliferation and migration of both TPC1 (**Figures 6D–H**) and BCPAP (**Supplementary Figures S4B-F**) cells. Finally, we used western blotting to validate that knockdown of NUP153 exhibited decreased expression of PI3Kγ, P-AKT-Ser^473^, and mTOR (**Figure 6I**, **Supplementary Figure S4G**).

### Downregulation of USB1 Promoted Tumor Growth and Migration *In Vitro*

**Figure 7:**
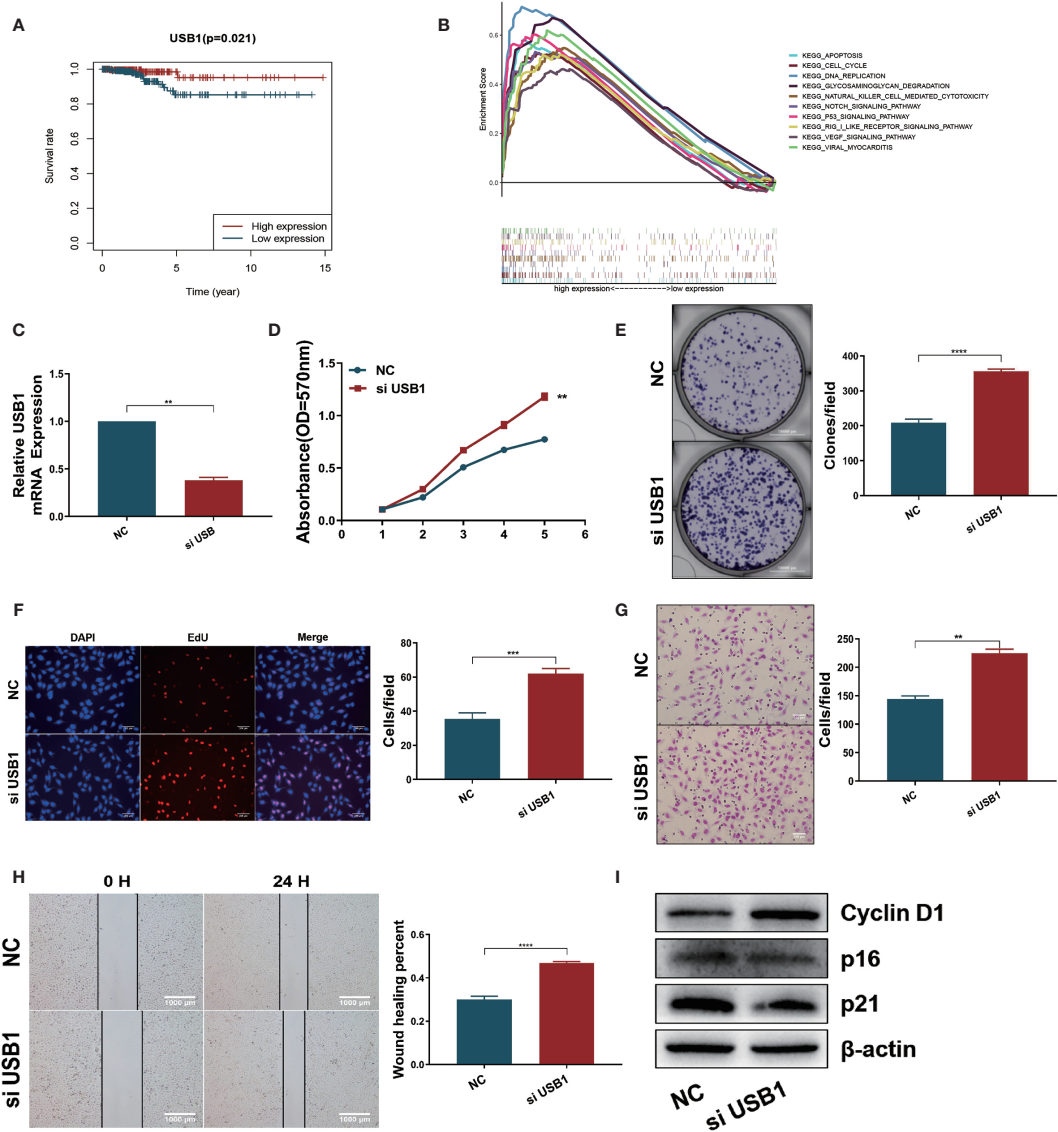
Downregulation of USB1 promoted tumor growth and migration *in vitro*. **(A)** High expression of USB1 was correlated with longer OS, according to Kaplan-Meier survival analysis in TCGA. Red, high expression. Blue, low expression. **(B)** Top ten significant enrichment results between USB1-high and USB1-low groups in THCA. **(C)** Real-time quantitative PCR for the expression of USB1 in NC and si-USB1 TPC1 cells. **(D-F)** MTT **(D)**, colony formation **(E)**, EdU **(F)** assays revealed cell growth promotion by downregulation of USB1 in TPC1 cells. **(G, H)** transwell **(G)** and wound healing/scratch **(H)** assays revealed increased migration of TPC1 cells following downregulation of USB1. **(I)** Downregulation of USB1 promoted the G1/S transition, as validated by western blot analysis. ****P < 0.0001, ***P < 0.001, **P < 0.01.

Survival analysis revealed that high expression of USB1 was associated with a good prognosis (**Figure 7A**), which was consistent with the results of the expression-heatmap (**Figure 4C**, bottom). GSEA was used to explore the biological signaling pathways underpinning the different outcomes between the high- and low-risk groups, and the cell cycle showed a significant enrichment score (NES = 1.69, NOM *p* = 0.033) (**Figure 7B**). We then carried out functional research by downregulating the expression of USB1 in TPC1 cells using a specific siRNA (**Figure 7C**). The downregulation of USB1 induced proliferation and migration in the TPC1 cells (**Figures 7D–H**). Furthermore, western blot analysis showed that knockdown of USB1 in TPC1 exhibited increased expression of cyclin D1 as well as decreased expression of p16 and p21 (**Figure 7I**), which indicated that the downregulation of USB1 promoted the G1/S phase transition of the cell cycle. To further confirm these results, the specific siRNA was used to knockdown USB1 expression in another thyroid cancer cell line, BCPAP (**Suplementary Figure S5**). As expected, USB1 depletion also increased cell proliferation and migration in BCPAP cells by promoting the G1/S transition.

## Discussion

With the increased use of diagnostic imaging and surveillance, thyroid cancer incidence continued to increase in many countries over the past decades. However, the newly diagnosed cases still account for a high proportion of cancer incidence, and patients with advanced thyroid carcinoma still have a poor 5-year survival rate. In recent years, increasing investigations focused on detecting genetic changes that can be used to diagnose thyroid cancer at an early stage to improve the patients’ 5-year survival rate. Several biomarkers have been applied in clinical practice, such as TfR1 activation of the ERK signaling pathway (22), PAX8-PPARγ rearrangement, as well as BRAF and RAS mutations (23, 24).

As a promising field of cancer biology, RNA-binding proteins (RBPs) have been shown to participate in the development of several types of malignant tumors. However, there was limited evidence of the involvement of RBPs in thyroid cancer. In this study, we extracted differentially expressed RBPs in thyroid cancer from the TCGA database. We found that these RBPs could regulate thyroid cancer through different biological processes and pathways. The functional enrichment analysis showed that the identified RBPs were mainly involved in RNA metabolic process, modification, translational regulation, splicing, localization and stabilization. Some RBPs involved in RNA associated processes have been reported. For instance, SNORD52 can promote the tumorigenesis of hepatocellular carcinoma (HCC) by enhancing the stability of CDK1 (25). YTH N6-methyladenosine RNA binding protein 1 (YTHDF1) was found to be positively associated with aggressive tumor progression and poor overall survival by promoting the translation of the essential Wnt receptor frizzled 7 (FZD7) in an m6A-dependent manner (26). In addition, APAK8-mediated alternative splicing produces the CLSTN1-S splice isoform, which inhibits EMT and shows an inverse correlation with breast cancer progression (27).

The prognostic model identified by multivariate Cox proportional hazards regression in this study was significantly associated with the overall survival of THCA patients (p = 0.009). Six genes were incorporated into this model, consisting of AZGP1, IGF2BP2, MEX3A, NUDT16, NUP153, and USB1. A recent research proved that AZGP1 is an AR target gene and is involved in androgen/AR axis-mediated cell proliferation and metastasis in primary PCa (28). Meanwhile the six-gene model revealed that high expression of AZGP1 was negatively associated with prognosis in thyroid cancer, so it might also play a critical role in thyroid cancer. A recent study identified that IGF2BP2 targets the lncRNA DANCR through m6A modification, and they work together to promote stemness and pathogenesis in pancreatic cancer (29). Furthermore, we noticed that high expression of IGF2BP2 was associated with a good prognosis in THCA patients, which implies that IGF2BP2 might also play an important role in thyroid cancer. The hydrolase activity of NUDT16 can remove ADP-ribosylation of 53BP1 to regulate its stability and localization at DNA double-strand breaks (DSBs) (30). MEX3A (mex-3 RNA binding family member A) was found to have an impact on intestinal differentiation, polarity and stemness, likely contributing to intestinal homeostasis and carcinogenesis *via* post-transcriptional regulation of CDX2 (31). Here, we found that MEX3A is deregulated in THCA patients based on the TCGA expression data. However, there is no direct experimental evidence showing that MEX3A participates in THCA progression. Taken together, the RBPs included in this model could play key roles in the development of different tumors, which merits further study.

In this study, we proved that nucleoporin 153 (NUP153), previously described as a critical factor in double-strand break repair and DNA damage response (32), acts as an oncogene in thyroid cancer by regulating the PI3K/AKT/mTOR pathway. U6 snRNA biogenesis phosphodiesterase 1 (USB1), also known as C16orf57, is mutated in poikiloderma with neutropenia (PN) and dyskeratosis congenita (DC) (33). In our study, we found that USB1 could induce cell cycle arrest in the G1 phase, thereby suppressing cell proliferation and migration. However, the mechanisms of these effects of NUP153 and USB1 need further study. Nevertheless, the results clearly show these six RBPs may have a prognostic value in THCA.

Overall, the integrated analysis of RBPs in thyroid cancer allowed us to construct a six-gene model associated with the overall survival of THCA patients. We identified that the prognostic genes are related to RNA degradation, transport, and catabolic processes. Moreover, experimental validation confirmed that NUP153 and USB1 impact the growth of thyroid tumor cell lines. Finally, the six-gene model might be applied as a potential prognostic signature in clinical practice in the future.

## Data Availability Statement

The gene expression data of 58 adjacent normal and 509 tumor tissue samples of thyroid cancer with corresponding clinical information were downloaded from the Cancer Genome Atlas (TCGA) database (https://portal.gdc.cancer.gov/).

## Author Contributions

YM and YY conceived and performed experiments, wrote the manuscript, and secured funding. YM, JH, and NC performed experiments. X-bZ and LF provided reagents. X-cC and YY provided expertise and feedback. All authors contributed to the article and approved the submitted version.

## Funding

This study was supported by the grants from the National Natural Science Foundation of China (No. 81502518 and No. 81702623).

## Conflict of Interest

The authors declare that the research was conducted in the absence of any commercial or financial relationships that could be construed as a potential conflict of interest. 

## References

[1] Bray F Ferlay J Soerjomataram I Siegel R Torre L Jemal A. Global cancer statistics 2018: GLOBOCAN estimates of incidence and mortality worldwide for 36 cancers in 185 countries. CA Cancer J Clin (2018) 6:394–424. 10.3322/caac.21492 30207593

[2] Cabanillas M McFadden D Durante C. Thyroid cancer. Lancet (London England) (2016) 10061:2783–95. 10.1016/s0140-6736(16)30172-6 27240885

[3] Cronin K Ries L Edwards B. The Surveillance, Epidemiology, and End Results (SEER) Program of the National Cancer Institute. Cancer (2014) 120:3755–7. 10.1002/cncr.29049 25412387

[4] Allemani C Weir H Carreira H Harewood R Spika D Wang X. Global surveillance of cancer survival 1995-2009: analysis of individual data for 25,676,887 patients from 279 population-based registries in 67 countries (CONCORD-2). Lancet (London England) (2015) 9972:977–1010. 10.1016/s0140-6736(14)62038-9 PMC4588097 25467588

[5] La Vecchia C Malvezzi M Bosetti C Garavello W Bertuccio P Levi F. Thyroid cancer mortality and incidence: a global overview. Int J Cancer (2015) 9:2187–95. 10.1002/ijc.29251 25284703

[6] Beckmann B Horos R Fischer B Castello A Eichelbaum K Alleaume A. The RNA-binding proteomes from yeast to man harbour conserved enigmRBPs. Nat Commun (2015) 6:10127. 10.1038/ncomms10127 26632259 PMC4686815

[7] Gerstberger S Hafner M Tuschl T. A census of human RNA-binding proteins. Nat Rev Genet (2014) 12:829–45. 10.1038/nrg3813 PMC11148870 25365966

[8] Lunde B Moore C Varani G. RNA-binding proteins: modular design for efficient function. Nat Rev Mol Cell Biol (2007) 6:479–90. 10.1038/nrm2178 PMC5507177 17473849

[9] Dreyfuss G Kim V Kataoka N. Messenger-RNA-binding proteins and the messages they carry. Nat Rev Mol Cell Biol (2002) 3:195–205. 10.1038/nrm760 11994740

[10] Mitchell S Parker R. Principles and properties of eukaryotic mRNPs. Mol Cell (2014) 4:547–58. 10.1016/j.molcel.2014.04.033 24856220

[11] Wurth L Papasaikas P Olmeda D Bley N Calvo G Guerrero S. UNR/CSDE1 Drives a Post-transcriptional Program to Promote Melanoma Invasion and Metastasis. Cancer Cell (2016) 5:694–707. 10.1016/j.ccell.2016.10.004 27908735

[12] Hopkins T Mura M Al-Ashtal H Lahr R Abd-Latip N Sweeney K. The RNA-binding protein LARP1 is a post-transcriptional regulator of survival and tumorigenesis in ovarian cancer. Nucleic Acids Res (2016) 3:1227–46. 10.1093/nar/gkv1515 PMC4756840 26717985

[13] Ishii H Saitoh M Sakamoto K Kondo T Katoh R Tanaka S. Epithelial splicing regulatory proteins 1 (ESRP1) and 2 (ESRP2) suppress cancer cell motility via different mechanisms. J Biol Chem (2014) 40:27386–99. 10.1074/jbc.M114.589432 PMC4183779 25143390

[14] Xu Y Gao X Lee J Huang H Tan H Ahn J. Cell type-restricted activity of hnRNPM promotes breast cancer metastasis via regulating alternative splicing. Genes Dev (2014) 11:1191–203. 10.1101/gad.241968.114 PMC4052765 24840202

[15] Lal A Kawai T Yang X Mazan-Mamczarz K Gorospe M. Antiapoptotic function of RNA-binding protein HuR effected through prothymosin alpha. EMBO J (2005) 10:1852–62. 10.1038/sj.emboj.7600661 PMC1142594 15861128

[16] Shen S Yang C Liu X Zheng J Liu Y Liu L. RBFOX1 Regulates the Permeability of the Blood-Tumor Barrier via the LINC00673/MAFF Pathway. Mol Ther Oncolytics (2020) 17:138–52. 10.1016/j.omto.2020.03.014 PMC7163051 32322670

[17] Gallardo M Lee H Zhang X Bueso-Ramos C Pageon L McArthur M. hnRNP K Is a Haploinsufficient Tumor Suppressor that Regulates Proliferation and Differentiation Programs in Hematologic Malignancies. Cancer Cell (2015) 4:486–99. 10.1016/j.ccell.2015.09.001 PMC4652598 26412324

[18] Puppo M Bucci G Rossi M Giovarelli M Bordo D Moshiri A. miRNA-Mediated KHSRP Silencing Rewires Distinct Post-transcriptional Programs during TGF-β-Induced Epithelial-to-Mesenchymal Transition. Cell Rep (2016) 4:967–78. 10.1016/j.celrep.2016.06.055 27396342

[19] Ritchie M Phipson B Wu D Hu Y Law C Shi W. limma powers differential expression analyses for RNA-sequencing and microarray studies. Nucleic Acids Res (2015) 7:e47. 10.1093/nar/gkv007 PMC4402510 25605792

[20] Shannon P Markiel A Ozier O Baliga N Wang J Ramage D. Cytoscape: a software environment for integrated models of biomolecular interaction networks. Genome Res (2003) 11:2498–504. 10.1101/gr.1239303 PMC403769 14597658

[21] Subramanian A Tamayo P Mootha V Mukherjee S Ebert B Gillette M. Gene set enrichment analysis: a knowledge-based approach for interpreting genome-wide expression profiles. Proc Natl Acad Sci U S A (2005) 43:15545–50. 10.1073/pnas.0506580102 PMC1239896 16199517

[22] Campisi A Bonfanti R Raciti G Bonaventura G Legnani L Magro G. Gene Silencing of Transferrin-1 Receptor as a Potential Therapeutic Target for Human Follicular and Anaplastic Thyroid Cancer. Mol Ther Oncolytics (2020) 16:197–206. 10.1016/j.omto.2020.01.003 32099899 PMC7033459

[23] Zou M Baitei E Alzahrani A BinHumaid F Alkhafaji D Al-Rijjal R. Concomitant RAS, RET/PTC, or BRAF mutations in advanced stage of papillary thyroid carcinoma. Thyroid (2014) 8:1256–66. 10.1089/thy.2013.0610 PMC4106383 24798740

[24] Barbie D Tamayo P Boehm J Kim S Moody S Dunn I. Systematic RNA interference reveals that oncogenic KRAS-driven cancers require TBK1. Nature (2009) 7269:108–12. 10.1038/nature08460 PMC2783335 19847166

[25] Li C Wu L Liu P Li K Zhang Z He Y. The C/D box small nucleolar RNA SNORD52 regulated by Upf1 facilitates Hepatocarcinogenesis by stabilizing CDK1. Theranostics (2020) 20:9348–63. 10.7150/thno.47677 PMC7415794 32802196

[26] Pi J Wang W Ji M Wang X Wei X Jin J. YTHDF1 promotes gastric carcinogenesis by controlling translation of FZD7. Cancer Res (2020). 10.1158/0008-5472.Can-20-0066 32788173

[27] Hu X Harvey S Zheng R Lyu J Grzeskowiak C Powell E. The RNA-binding protein AKAP8 suppresses tumor metastasis by antagonizing EMT-associated alternative splicing. Nat Commun (2020) 1:486. 10.1038/s41467-020-14304-1 PMC6981122 31980632

[28] Cao R Ke M Wu Q Tian Q Liu L Dai Z. AZGP1 is androgen responsive and involved in AR-induced prostate cancer cell proliferation and metastasis. J Cell Physiol (2019) 10:17444–58. 10.1002/jcp.28366 30820960

[29] Hu X Peng W Zhou H Jiang J Zhou X Huang D. IGF2BP2 regulates DANCR by serving as an N6-methyladenosine reader. Cell Death Differ (2020) 6:1782–94. 10.1038/s41418-019-0461-z PMC7244758 31804607

[30] Zhang F Lou L Peng B Song X Reizes O Almasan A. Nudix Hydrolase NUDT16 Regulates 53BP1 Protein by Reversing 53BP1 ADP-Ribosylation. Cancer Res (2020) 5:999–1010. 10.1158/0008-5472.Can-19-2205 PMC7056578 31911551

[31] Pereira B Sousa S Barros R Carreto L Oliveira P Oliveira C. CDX2 regulation by the RNA-binding protein MEX3A: impact on intestinal differentiation and stemness. Nucleic Acids Res (2013) 7:3986–99. 10.1093/nar/gkt087 PMC3627580 23408853

[32] Lemaître C Fischer B Kalousi A Hoffbeck A Guirouilh-Barbat J Shahar O. The nucleoporin 153, a novel factor in double-strand break repair and DNA damage response. Oncogene (2012) 45:4803–9. 10.1038/onc.2011.638 22249246

[33] Mroczek S Krwawicz J Kutner J Lazniewski M Kuciński I Ginalski K. C16orf57, a gene mutated in poikiloderma with neutropenia, encodes a putative phosphodiesterase responsible for the U6 snRNA 3’ end modification. Genes Dev (2012) 17:1911–25. 10.1101/gad.193169.112 PMC3435495 22899009

