# Supplementary material for: Comprehensive Analysis of the Functions and Prognostic Value of RNA-Binding Proteins in Thyroid Cancer
Source: Front Oncol. 2021 Mar 17;11:625007. doi: 10.3389/fonc.2021.625007 (PMC8010172; doi:10.3389/fonc.2021.625007)
Supplement: Supplementary file 1 [file DataSheet_1.pdf]

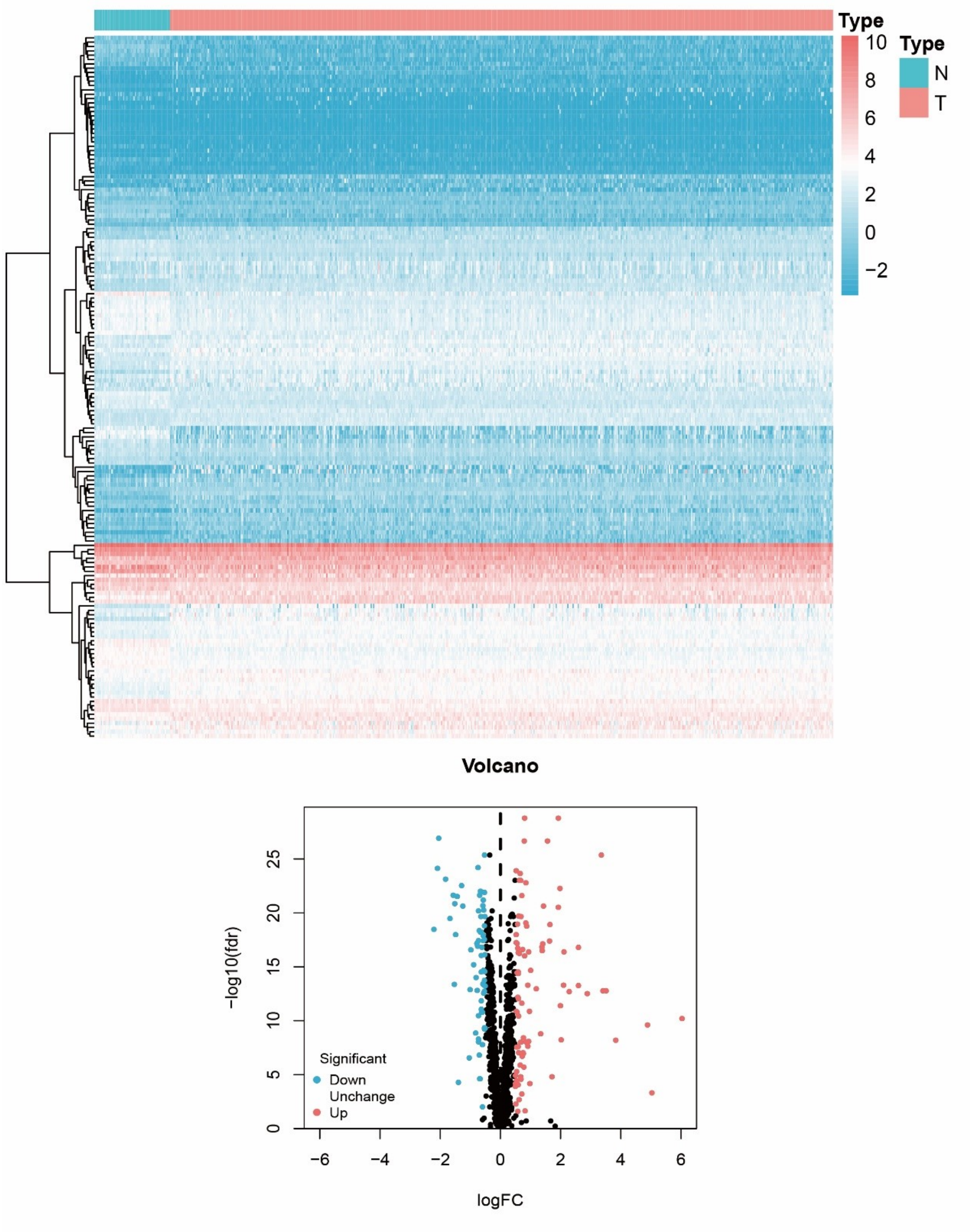

Figure S1. The expression distribution of differentially expressed RBPs. (A) The heatmap of 162 differentially expressed RBPs. (B) The volcano plot of differentially expressed RBP. Red, upregulated. Blue, downregulated.

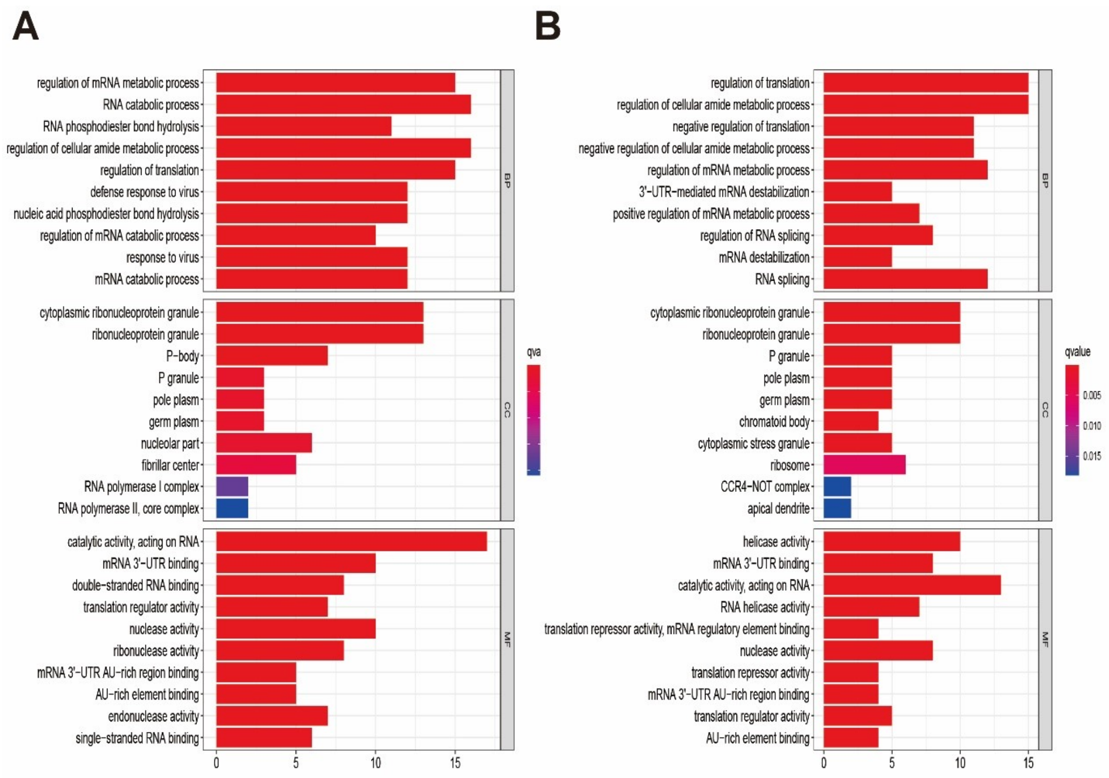

Figure S2. GO analyses of differentially expressed RBPs. (A) Top ten categories of GO analysis result of upregulated RBPs. (B) Top ten categories of GO analysis result of downregulated RBPs. BP, biological processes. CC, cellular component. MF, molecular function.

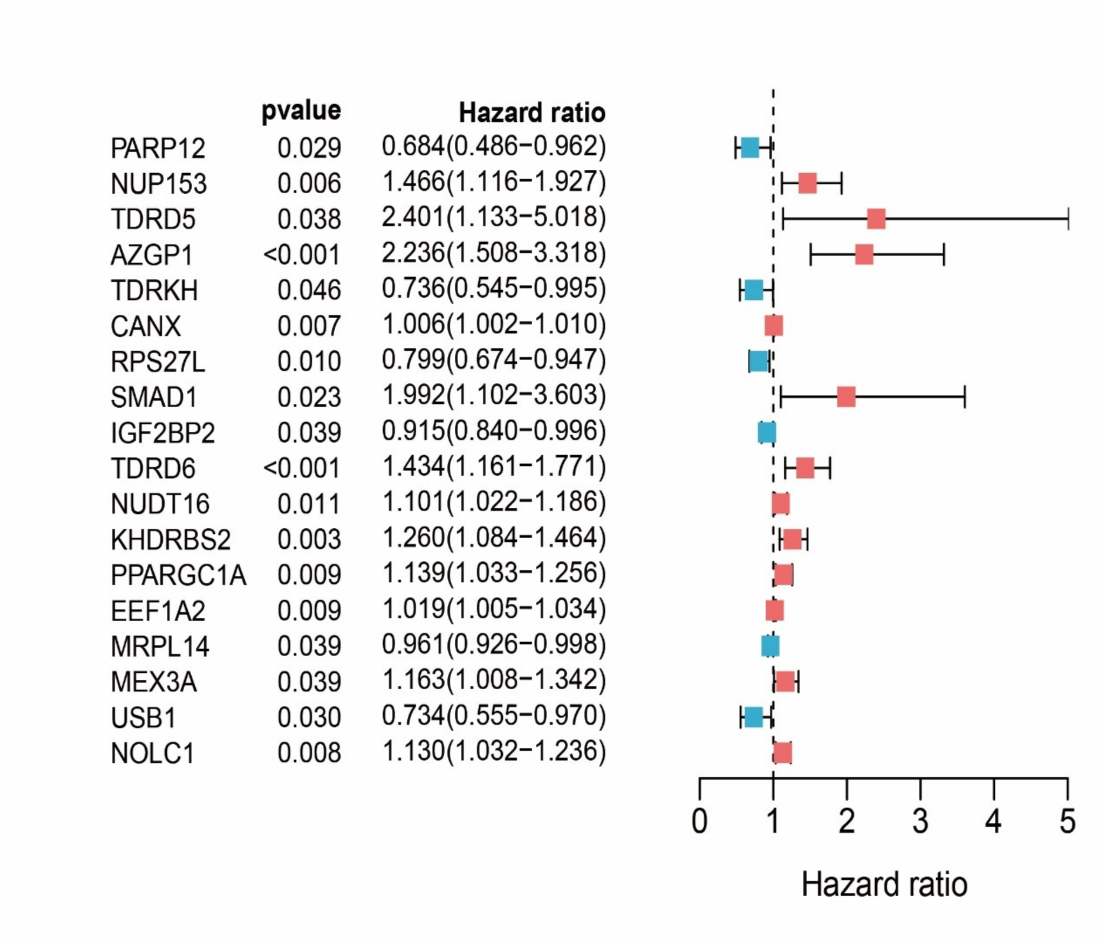

Figure S3. The forest plot of prognostic value of prognosis-related RBPs. Red, protooncogenic RBPs. Blue, tumor-suppressed RBPs.

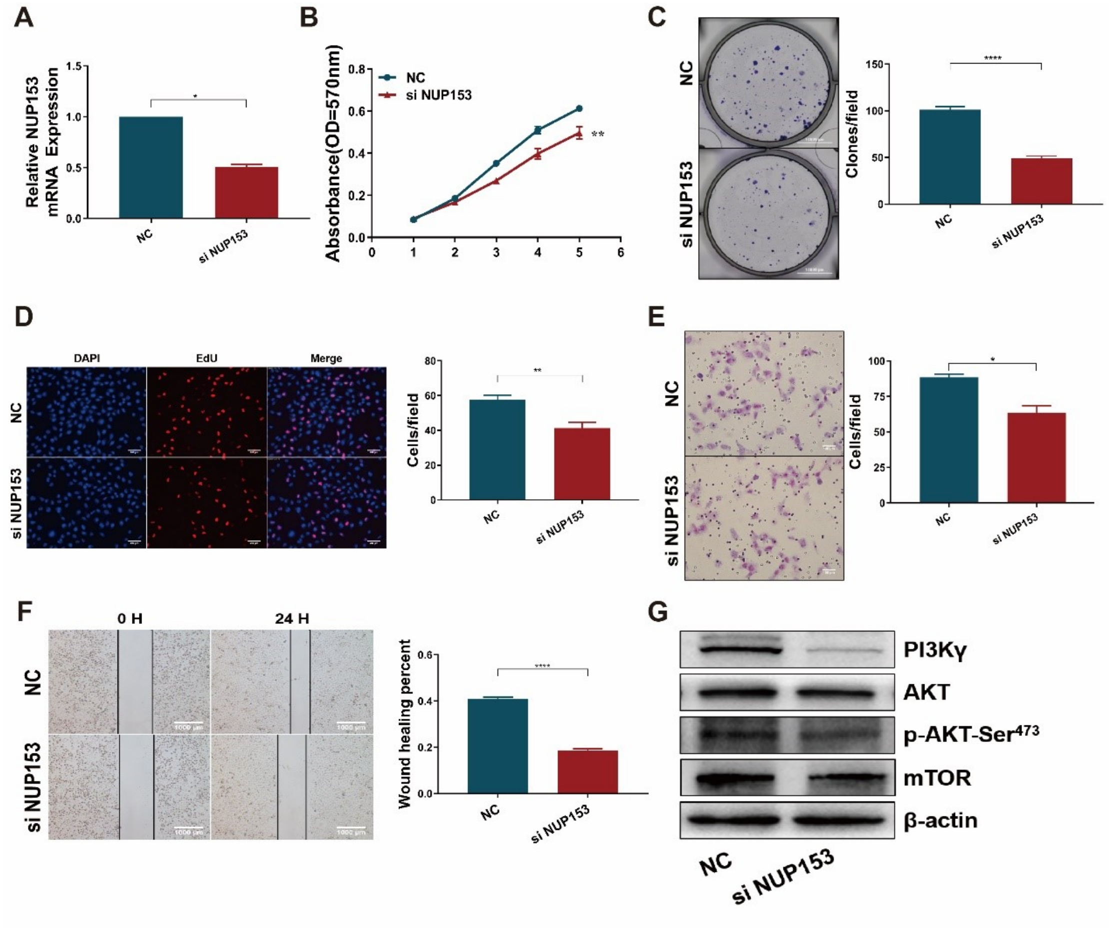

Figure S4. Downregulation of NUP153 inhibited proliferation and migration of the BCPAP cell. (A) Real-time quantitative PCR for the expression of NUP153 in different groups of BCPAP cell. **(B-D)** Growth inhibition in BCPAP cell was determined by MTT (B), colony formation (C) and EdU (D) assay. **(E, F)** Cell migration inhibition was determined by transwell (E) and wound healing/scratch (F) assay. **(G)** Western blot validation showed the decreased expression of PI3Kγ, P-AKT-Ser^{473}, mTOR. ****P < 0.0001, **P < 0.01, *P < 0.05

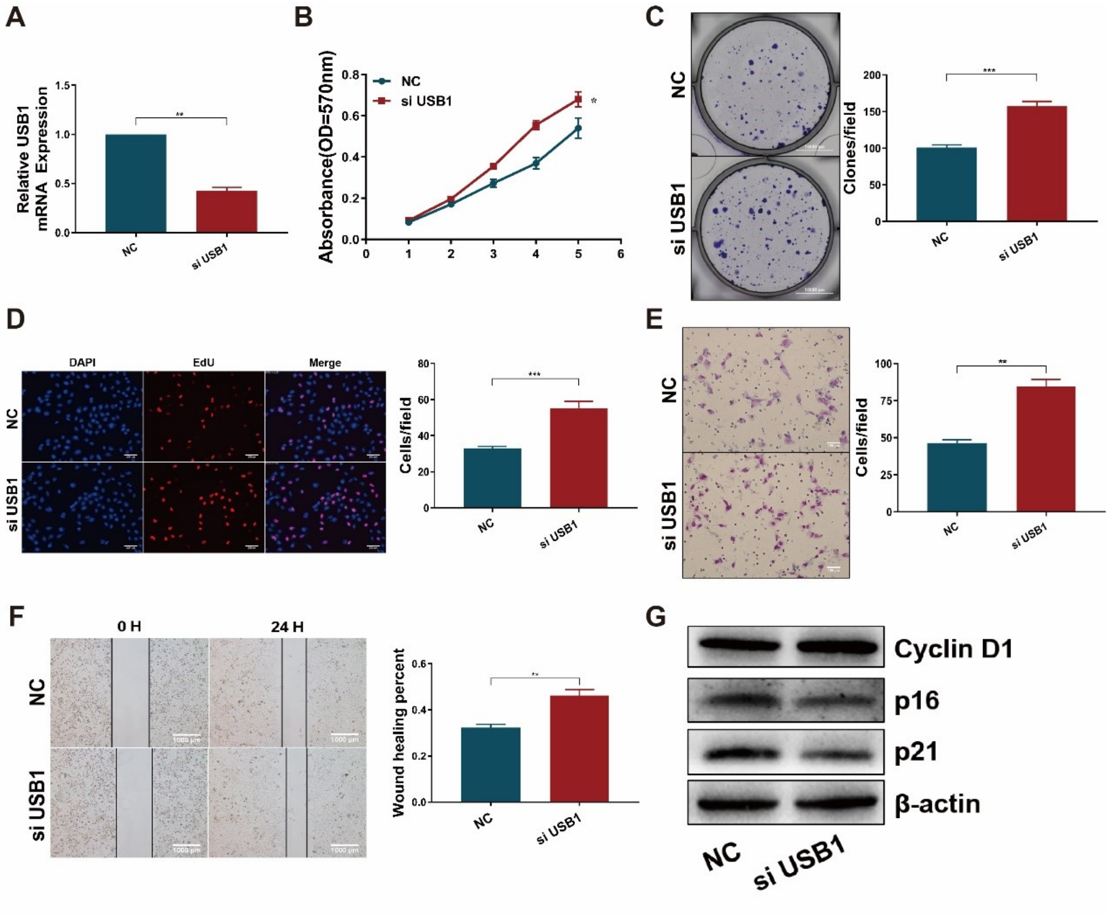

Figure S5. Downregulation of USB1 promote tumor growth and migration in BCPAP cell. (A) Real-time quantitative PCR for the expression of USB1 in NC and si-USB1 BCPAP cell. (B-D) MTT (B), colony formation (C), EdU (D) assay revealed the cell growth promotion by downregulation of USB1 in BCPAP cell. (E, F) transwell (E) and wound healing/scratch (F) assay revealed increased migration of downregulated USB1 in BCPAP cell. (I) Downregulation of USB1 promoted G1 / S transition validated by western blot. ***P < 0.001, **P < 0.01, *p<0.05

Table S3. Primers used for RT-qPCR

Genes	Forward (5'-3')	Reverse (5'-3')
NUP153	CAGGGGCCAATTAAGCCTTAC	ACCTCGCTTGTGTCTGTTGAA
USB1	ATGAGTCCGAGGACGGGATG	TGTTCAGCACACTGTCAGGTA
GAPDH	ACAACTTTGGTATCGTGGAAGG	GCCATCACGCCACAGTTTC

Table S4. Target sequence of siRNA

ID	Gene name	sense（5'-3'）	antisense（5'-3'）
si NUP153	NUP153 (human)	GTACCTGCTTCAAGTAGCA	TGCTACTTGAAGCAGGTAC
si USB1	USB1 (human)	CACCCACGTCTATGTACCA	TGGTACATAGACGTGGGTG

Supplementary materials and Mathods

Antibodies and small interfering RNA (siRNA)

Antibodies against AKT, P-AKT-Ser^{473}, mTOR, PI3Kγ, Cyclin D1, p16, p21 and β-actin (Cell Signaling Technology, Beverly, MA, USA) were used. siRNAs targeting NUP153 and USB1, and non-specific control siRNA were purchased from RiboBio (Guangzhou, China). The specific target sequences were listed as Table S4.

Proliferation assay

Both MTT, plate colony formation and EdU assays were used to evaluate the capacity of cell proliferation. For MTT assay, 48 h after transfection, cells were seeded into 96-well plates at a density of 1.5×10^3 cells/per well. After incubation for the indicated time, cells were incubated with 20 μl MTT (0.5 mg/mL; Beyotime Institute of Biotechnology) at 37 °C for 1 h. The absorbance at 570 nm was detected using a microplate auto-reader (Bio-Tek Elx 800, USA).

For plate colony formation assay, 48 h after transfection, cells were seeded in 6-well plates at a density of 500 cells/per well. About 2 weeks later, the clones were washed with phosphate buffered saline (PBS) and fixed by 4% fixative solution for 30 min at room temperature and then washed with PBS, followed by staining with 1% crystal violet solution. The number of clones were counted and compared with control.

The EdU assay was detected by EdU labeling/detection kit (Ribobio) according to the manufacturer's protocol. Briefly, after transfection for 48 h, cells were incubated with 50μM EdU for 3 h. The cells were fixed with 4% formaldehyde for 30 min at room temperature. After being washed with PBS, cells were reacted with Apollo reaction cocktail for 30 min and treated with 0.5% Triton X-100 for 15 min at room temperature for permeabilization. Subsequently, cell nuclei were stained with Hoechst 33342 at a concentration of 0.1μg / ml for 30 min. The cells were then observed under a fluorescence microscope. The percentage of EdU-positive cells was examined by fluorescence microscopy.

Migration assay

The migration performance of THCA cells in vitro were evaluated by Matrigel-coated Transwell (BD Biosciences, San Diego, CA, USA) and wound healing / scratch assay. Briefly, medium containing 20% FBS was added to the lower chamber, and 3×10^4 cells in 200 μl serum-free medium were added to the upper chamber. About 12 hours later, the migrant cells were fixed and stained with THREE-STEP STAIN SET (Thermo, USA) according to the manufacture's instruction. The membranes were then carved and embedded under coverslips with the cells upward. The number of migrating cells was counted under a microscope in five predetermined fields.

For wound healing / scratch assay, cells were seeded into 6-well plates. After 24 h,

similar straight scratches have been made on monolayers in each well using 20 μl sterile pipette tips and washed twice with PBS and cultured with serum-free medium. At 0 h, 24 h, and 48 h time points, two random pictures under a microscope (at 4× magnifications) were taken for each wound. The areas between the edges of the wounds have been measured and analyzed using Adobe Photoshop software.
